# Leveraging Algorithms to Improve Decision-Making Workflows for Genomic Data Access and Management

**DOI:** 10.1089/bio.2022.0042

**Published:** 2022-10-17

**Authors:** Vasiliki Rahimzadeh, Jonathan Lawson, Greg Rushton, Edward S. Dove

**Affiliations:** ^1^ Stanford Center for Biomedical Ethics, Stanford University, Stanford, California, USA.; ^2^ Broad Institute of MIT and Harvard, Cambridge, Massachusetts, USA.; ^3^ School of Law, University of Edinburgh, Edinburgh, United Kingdom.

**Keywords:** automated decision system, data access committee, ethics, genomics, health

## Abstract

Studies on the ethics of automating clinical or research decision making using artificial intelligence and other algorithmic tools abound. Less attention has been paid, however, to the scope for, and ethics of, automating decision making within regulatory apparatuses governing the access, use, and exchange of data involving humans for research. In this article, we map how the binary logic flows and real-time capabilities of automated decision support (ADS) systems may be leveraged to accelerate one rate-limiting step in scientific discovery: data access management. We contend that improved auditability, consistency, and efficiency of the data access request *process* using ADS systems have the potential to yield fairer *outcomes* in requests for data largely sourced from biospecimens and biobanked samples. This procedural justice rationale reinforces a broader set of participant and data subject rights that data access committees (DACs) indirectly protect. DACs protect the rights of citizens to benefit from science by bringing researchers closer to the data they need to advance that science. DACs also protect the informational dignities of individuals and communities by ensuring the data being accessed are used in ways consistent with participant values. We discuss the development of the Global Alliance for Genomics and Health Data Use Ontology standard as a test case of ADS for genomic data access management specifically, and we synthesize relevant ethical, legal, and social challenges to its implementation in practice. We conclude with an agenda of future research needed to thoughtfully advance strategies for computational governance that endeavor to instill public trust in, and maximize the scientific value of, health-related human data across data types, environments, and user communities.

## Introduction

Human health research frequently involves the collection, use, and exchange of identifiable data and thus requires additional oversight. To ensure compliant use of identifiable data, data access committees (DACs), institutional review boards, and other oversight bodies work in concert to review requests for data and report on its usage. Specifically, DACs assess whether the proposed research uses of data comply with the participant consent and data access agreements that govern their use. However, the increasing volume of and real-time demand for genomic data are exceeding the capabilities of manual DAC review.^1^

Opaque decision making, inconsistent or duplicative decisions when data access requests are reviewed by multiple DACs, and other process inefficiencies are growing concerns that risk responsible governance of data derived principally from biospecimens and biobanked samples.^2^ Therefore, delays and inadequacies in DAC review directly impact the expeditious and compliant use and distribution of biobank samples to advance biomedical research, and they impact human health.^3–5^

In this article, we explore the advantages and limitations of automated decision support (ADS) systems—a coordinated system of algorithms, software, and ontologies—to facilitate human data access management.^6^ ADS systems could help standardize and expedite review procedures, yielding fairer, more consistent, and timelier DAC review outcomes, and subsequently timelier researcher access to data. The improved auditability, consistency, and efficiency afforded by ADS systems can drive greater parity between data access review processes and outcomes.

We interrogate how ADS may supplement, without replacing, the central roles that human reviewers play in data access adjudication. We discuss the development of an open-source standard and corresponding software, the Global Alliance for Genomics and Health (GA4GH) Data Use Ontology (DUO) (https://github.com/EBISPOT/DUO) and Data Use Oversight System (DUOS) (https://duos.broadinstitute.org/), respectively, as an ADS test case for semi-automating genomic data access management. Next, we synthesize relevant ethical, legal, social, and technical considerations that ought to precede any possible ADS implementation for managing access to diverse data types, across multiple data environments, and among various user communities.

We advance a procedural justice argument invoked namely in the resource allocation literature^7^ to justify why investing in the development, implementation, and ongoing evaluation of ADS should be pursued for the effective management of genomic and health-related data resources across the research lifecycle. Finally, we conclude with an agenda of future research needed to determine the efficacy of ADS-enabled strategies for access management in the biobanking and genomic data science space, and, importantly, to evaluate the potential impact that such systems may have on public willingness to share their samples and data moving forward.

## Algorithms: A Brief Primer

**FIG. 1.**
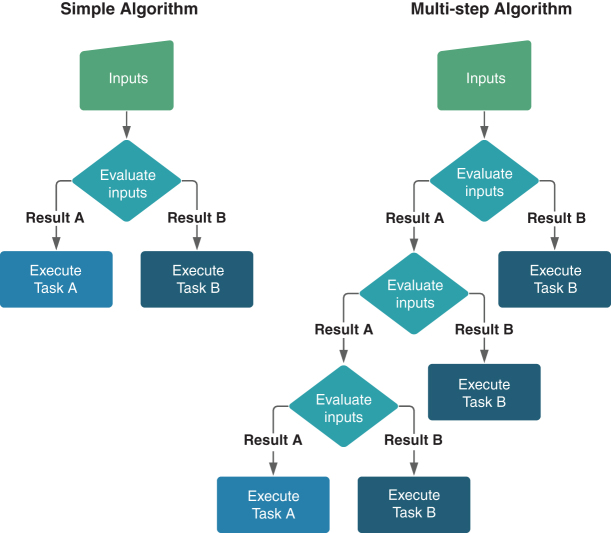
Prototypical logic flow of simple and complex algorithms.

All algorithms involve a sequence of preprogrammed steps that a computer follows to execute a given task. Computer scientists agree on little else regarding a formal definition of algorithms beyond this most basic feature, though rich debates abound.^8–11^ We adopt the liberal definition proposed by Cormen et al. that an algorithm is, “informally, any well-defined computational procedure that takes some value, or set of values, as input and produces some value, or set of values, as output.”^12^ In their simplest forms, algorithms comprise a singular or series of “if/then” statements that trigger some desired function when an “if” condition is met (Fig. 1).

An algorithm's complexity increases with every additional “if/then” statement, as it creates new circumstances to which the computer must execute a task. Machine learning typifies the most complex algorithms because they may refine evaluation criteria for inputs without explicit direction from their human architects. Moreover, machine-learning algorithms calibrate functional outputs to optimize outcomes based on preset parameters. In nonmachine learning algorithms, humans predetermine the logical steps that an algorithm takes and retain sovereign control over inputs and outputs.

Therefore, elements of a decision workflow that are best suited to ADS are those that involve binary “if” determinations that correspond to a discrete set of “then” actions and that can be made machine-readable. This is because ADS are programmed to deliver consistent outputs based on user-identified inputs and stable functions. They can reduce if/then classification errors that humans are statistically more prone to make, conferring one potential advantage in terms of consistency.

The ADS systems could promote consistency not only within a DAC over time, but they are also likely able to bring uniformity across DACs if implemented in a federated system such as DUOS. Moreover, ADS systems enable auditability across the decision-making and execution pathways. Such transparent audit mechanisms are rarely, if ever, realized among human DACs because most decisions are not publicly available.

At a minimum, these advantages warrant exploring whether ADS systems could enhance compliance and reporting aspects of the data access management process where transparency and consistency are highly valued. Scarce human resources could then more effectively be allocated to address complex applications for data access, which may necessitate deeper ethical deliberation. Such issues may include determining fringe cases in which proposed data uses are not easily machine-readable, when prospective applicants lack institutional credentials (for example, citizen scientists) or when proposed uses could invite community-based harms.

## Case Study: The DAC

### Genomic data generation, access management, and the problem of manual user verification

Collection and sharing of genomic and health-related data drives discovery in precision medicine. However, data are being generated at a pace and in volumes that overwhelm the ability of many institutions to enable their secure access.^13,14^ Both unjustifiably restrictive and overly permissive access to data can violate core principles of research ethics as well as the human right to benefit from science.^15^ Overly restricting data access prevents researchers from working with data they would otherwise be ethically and legally permitted to use to rigorously test scientific hypotheses and reproduce analyses. This can result in data waste and un(der)utilization. Conversely, granting access to data without verifying user identities heightens risks for data misuse, especially given the ease with which individuals can be re-identified.^16^ This compromises public trust.

DACs, therefore, serve as key institutional data stewards who evaluate access requests using criteria that balance protection of the rights and interests of data subjects with facilitating research endeavors and innovation through appropriate data use.^17,18^ They often consist of one or more members who may oversee multiple distinct datasets or repositories. DAC members may receive requests from researchers within, as well as external to, institutions, sectors, and countries. Empirical research suggests that the primary roles and administrative duties of DACs are largely conserved,^19^ namely ensuring the:

 • Proposed research is within the bounds of the data's permitted uses (i.e., is the data *use* appropriate?)
 • Researcher is qualified and permitted to access the data (i.e., is the data *requester* appropriate?)
 • Researcher has obtained required regulatory and ethics review approvals (i.e., is the data *project* appropriate?)

Despite these similarities, no procedural standards guide how DACs operate consistently, compliantly, and efficiently.^17^ Such a lack of procedural standardization delays the time from initial request to authorization, can lead to inconsistent DAC review decisions and, ultimately, compromises data protections.

Precisely calibrated data access procedures are, therefore, critically needed to maximize the speed and ethical sourcing of participant data for genomic discovery.^20^ Extant procedures for managing access continue to rely on static approaches to data generation and manual verification of use permissions. These approaches are rate-limiting factors dampening the potential of the transition to cloud-native data commons and insufficient to meet the high demand for dynamic, real-time data access.^21^ Previous research confirms that manual data access management systems constrain DACs' abilities to review requests in high volumes,^5^ whereas the predominance of manual access controls complicates oversight and data use compliance with participant consent and data access agreements.^2^ Establishing a truly borderless computing environment in the cloud also remains a challenge due to jurisdiction-specific requirements for data security and protection.^22^

### Development and beta testing of the DUO standard for genomic data access adjudication

To expedite the compliant human-administered management of data access and sharing, the GA4GH developed the DUO. The DUO advanced prior foundational work on applying semi-automated approaches to data access management, namely by introducing machine-readable data use terms.^23,24^ The DUO is a machine-readable, structured vocabulary of terms and definitions that describe consented data uses often found in informed consent forms.^25^

Recently, the DUO has been implemented in the Broad Institute's DUOS, an open-source software platform (https://duos.org/) with various services to support DACs and data sharing. The DUOS uses the GA4GH DUO standard to codify consented data uses, which are the key inputs of the data access decision managed by DACs. By codifying these inputs, DUOS can present standardized data use terms to human-led DACs. The DUOS then inputs the machine-readable terms into a semantic reasoning algorithm that attempts to mirror human DAC decisions.

**FIG. 2.**
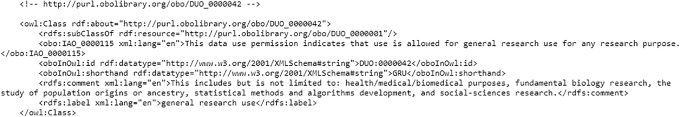
Examples of datasets tagged with DUOS terms. DUOS, Data Use Oversight System.

**FIG. 3.**
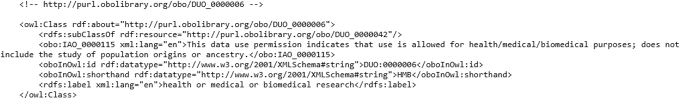
Examples of datasets tagged with DUOS terms.

Every new dataset created in DUOS is tagged with DUO terms (Figs. 2 and 3). Researchers assign the appropriate DUO terms to describe the nature of their data access request, for example, http://purl.obolibrary.org/obo/DUO_000006, the DUO term for health, medical, or biomedical research use. Structurally, DUO_000006 is a subclass of DUO_000042 and therefore is a semantically valid child of DUO_000042. Using traditional ontology reasoning tools (see, e.g., https://github.com/owlcs/owlapi and https://github.com/DataBiosphere/consent-ontology/blob/develop/docs/UseRestrictionGrammar.md), we can infer that any research purpose tagged with DUO_000006 is a semantic “match” for any dataset tagged with DUO_0000042, and given no other inputs would lead DUOS algorithm to approve the request.

Using this technique, DUOS can allow users to combine multiple ontology terms, thereby constructing more complex inputs that standard Ontology reasoning engines can logically parse to determine whether the researchers' data access requests are within the bounds of the datasets data use permissions.

Figures 2 and 3 display the full description of the DUO terms for General Research Use and Health, Medical, Biomedical research and are available with all other DUO terms at https://raw.githubusercontent.com/EBISPOT/DUO/master/duo.owl

In practice, such a tool can be used either as a decision support tool—operating concurrently with the DAC and providing a suggested decision—or as the central decision maker, which affords the DAC opportunities for efficiency wins in automation. DUOS currently leverages its algorithm in the decision support tool format. Moreover, in a recent head-to-head comparison of 51 data access requests, the DUOSs algorithm concurred with the human DACs decision in all 51 cases.^26^

### Implementation challenges

The same disruptive attributes that make ADS compelling for data access management are also those that pose challenges for effective implementation and evaluation. First, building and transitioning to an ADS system is resource-intensive, requiring both material (e.g., software, hardware) and human investment (e.g., developers, user experience experts, scientists, lawyers). This would include adequate training for DAC members regarding how DUOS was developed as well as how it could be integrated into existing DAC workflows to optimize review processes.

Second, ADS can require a substantial overhaul of existing workflows and decision-making processes or policies that may have cemented within institutions over time. Second, decisions worth the investment of converting to ADS typically carry nonzero liability. Substantial buy-in from internal and external stakeholders is needed to assess organizational readiness, determine risk-benefit trade-offs, and manage the transition from reliance on manual procedures to ADS. Third, and more specifically, evaluation of ADS in the data governance context is severely limited to date.

To our knowledge, no research yet demonstrates what impacts ADS are likely to have on public willingness to share their data nor how ADS may affect trust in the institutions responsible for data stewardship. Both are critical to responsible ADS deployment, and they are the foci of ongoing empirical work on ADS implementation barriers and facilitators partnership with DAC members worldwide. We have reason to believe, however, that prospective data contributors are cautious of ADS given the rise in public consciousness of algorithmic fairness, discrimination, and surveillance implicated in health care applications of ADS, particularly among marginalized communities.^27–29^

Understanding the complexities and hurdles to adoption, the DUOS team intentionally leaves its code open source, produces and publishes results on the progress of the algorithm's fidelity with human decisions, and remains available to advise others seeking to implement similar tools.

## ADS for Data Access Management

### Normative justifications and practical considerations

Notwithstanding the earlier mentioned challenges and the need for a more robust research agenda into questions of algorithmic fairness and equity, there are strong procedural justice reasons for supporting hybridized human-ADS approaches to manage data access. Drawing on the Rawlsian tradition, procedural justice relates the fairness perceived in how resources are distributed to the outcomes resulting from that resource distribution. The DAC review could be considered procedurally fair when the following conditions are met:

I. Independent criteria exist to determine just outcomes of the procedure—that is, access to data is granted when the data user, data use, and data type cohere with permissions outlined in participant consent and comply with data access agreements; and the
II. Procedure guarantees that the fair outcomes will be achieved—only authorized researchers access data for approved purposes.

Condition (I) is predicated on social consensus about the rules, norms, and laws governing responsible data access and that are subject to change in the wake of technological innovations, regulatory reforms, or evidence of social harm. ADS activates the procedural integrity required to meet condition (II), complying with the independent criteria with perfect accuracy. To this end, we identify six technical safeguards unique to ADS systems and propose what normative value they bring to the data access review and management process in Table 1.

**Table 1.** Technical Safeguards Unique to Automated Decision Support Systems and Their Normative Value-Add to the Data Access Review and Management Process

Decision system attribute	Normative value-add
Federated capability	ADS can be applied uniformly across databases, avoiding duplication, and improving allocation of finite human resources. Global or widespread endorsement of a policy by which such an algorithm operates allows any participating groups to leverage the same efficiency benefits, while also providing consistency of decision making across institutions and countries.
Real-time task execution	Elements of the adjudication process, once made machine readable (e.g., user authentication, verification, data use permissions), allow algorithms to make dynamic decisions and execute actions in real time, improving review efficiency to a potentially instantaneous turnaround.
Adaptation	Any element of the ADS workflow can be modified without special effort at any time, and the effects of these modifications can be experienced immediately. This affords more granular compliance capability that will become increasingly important as the regulatory landscape of data protection becomes more complex.
Audit capability	All prior and current versions of the source for ADS can be made publicly available on a subscription-fee database, enabling transparency and auditability of the software by community members.
Binary logic flow	The consistent series of if/then logic flows ensures equivalent inputs are treated equally and result in identical outputs, enabling greater consistency and transparency in review decisions, such as permitted, permitted with restrictions, or denied.
Record tracking	Comprehensive records of all human and machine-readable inputs (request for access) and outputs (access review decisions) may be kept. This enables greater accountability to stakeholders affected by DAC decisions, notably data users and stewards, and provides opportunities to appeal review decisions.

ADS, automated decision support; DAC, data access committee.

**FIG. 4.**
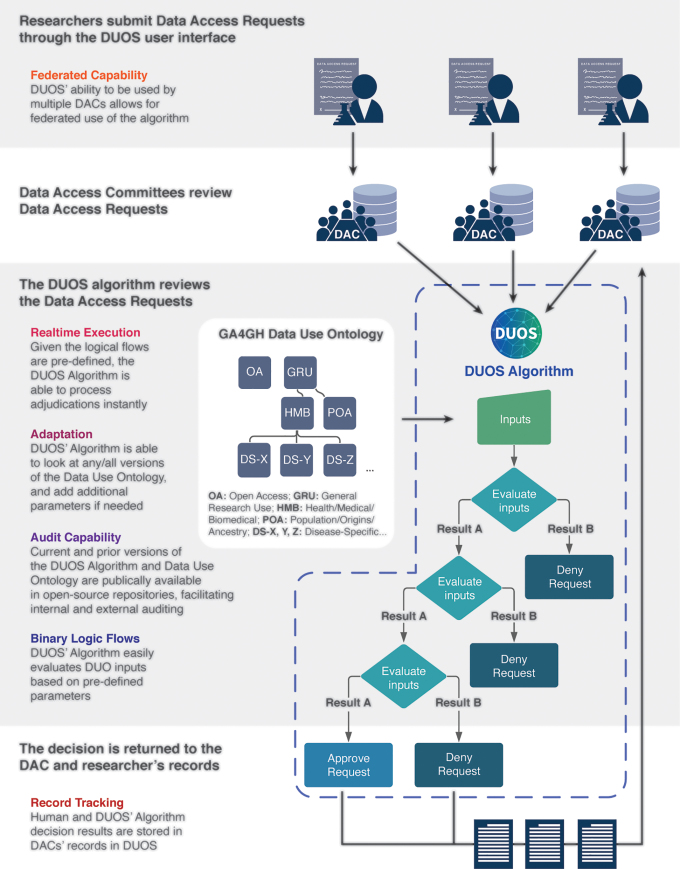
Real-world data access request using DUOS software highlighting how attributes of automated decision support systems could be leveraged to improve DAC review quality and effectiveness. DAC, data access committee.

We contend that ADS could help human DACs better realize procedural justice goals that are the most critical to a well-functioning data access review management system in the life sciences, including consistency, quality, effectiveness, and efficiency. This is further illustrated in Figure 4, which maps a real-world data access request using DUOS and highlights how ADS attributes are leveraged to improve DAC review quality and effectiveness, among other outcomes.

While purposed with genomic data sharing in mind, the DUO codifies general secondary data use permissions. It could, thus, easily represent secondary use terms for myriad scientific data types and biospecimens. Indeed, we are aware of at least one biobank that employs DUO terms to facilitate sample or biospecimen access.^25^ We envision a future in which a variety of biobanks adopt DUO and ADS software such as DUOS to meet their specific workflow needs.

Future DUO users should not face implementation challenges with respect to complying with specific data protection regulations, such as the EU's General Data Protection Regulation (GDPR) since DUO does not seek to represent GDPR or other regulatory terms but rather terms outlined in participants' consent. Therefore, the DUO (and related ADS such as DUOS) can be applied and transferred to biobanks situated across the globe as a support to current data protection regulation-related compliance procedures, without concern for increasing or complicating the compliance burden.

## Conclusion

Incorporating ADS into human-led decisional workflows is a promising approach for making the process of data access decision making and oversight more effective, consistent, and efficient. At least six technical attributes motivate a computational approach to data access management, including federation, real-time task execution, adaptation, audit, binary decision making, and record tracking. Taken together, ADS have potential to enhance procedural justice in data access review and instill greater confidence that the decisions made by DACs are defensible, fair, and consistent from the perspectives of key stakeholders in health research, namely researchers, participants, and society.

Effective ADS implementation is currently limited by little empirical data on the organizational readiness of DACs to adopt (semi)automated workflows, public skepticism of algorithmic fairness and bias, training, and the resource intensiveness of building digital infrastructures to support software-mediated reviews, among others. Future research is needed to assess the ethical, legal, and social issues associated with transitions to computation-based data governance mediated by algorithms and other decision support tools. Toward this end, we encourage periodic algorithmic impact assessment early in the ADS development and implementation stages as recently proposed by fellow data ethicists,^30,31^ as well as empirical investigation into relevant opportunities and barriers to ADS implementation in data access and biospecimen management.

## Related Links

The Global Alliance for Genomics and Health's Data Use Ontology is available via https://github.com/EBISPOT/DUO. The DUOS algorithm description is available at https://github.com/DataBiosphere/consent-ontology/blob/develop/docs/UseRestrictionGrammar.md. Documentation for the OWL reasoner is available at https://github.com/owlcs/owlapi
